# The Effect of Paxilline on Early Alterations of Electrophysiological Properties of Dentate Gyrus Granule Cells in Pilocarpine-Treated Rats 

**Published:** 2014

**Authors:** Nasrin Mehranfard, Hamid Gholamipour-Badie, Fereshteh Motamedi, Mahyar Janahmadi, Nima Naderi

**Affiliations:** a *Department of Pharmacology and Toxicology, School of Pharmacy, Shahid Beheshti University of Medical Sciences, Tehran, Iran.*; b *Neuroscience Research Center, Shahid Beheshti University of Medical Sciences, Tehran, Iran. *; c *Department of Physiology, Faculty of Medicine, Shahid Beheshti University of Medical Sciences, Tehran, Iran. *

**Keywords:** Paxilline, Dentate gyrus, Granule cells, Epilepsy

## Abstract

The dentate gyrus of hippocampus has long been considered as a focal point for studies on mechanisms responsible for the development of temporal lobe epilepsy (TLE). Change in intrinsic properties of dentate gyrus granule cells (GCs) has been considered as an important factor responsible in temporal lobe seizures. In this study, we evaluated the intrinsic properties of GCs, during acute phase of seizure (24 h after *i.p*. injection of pilocarpine) compared to sham group using whole cell patch-clamp recordings. Our results showed a significant increase in the number of action potentials (APs) after applying depolarizing currents of 200 pA (p < 0.01) and 250pA (p < 0.05) compared to sham group. The evaluation of AP properties revealed a decrease in half-width of AP in GCs of seizure group (1.27 ± 0.03 ms) compared to sham group (1.60 ± 0.11). Moreover, addition of BAPTA to pipette solution prevented changes in AP half-width in seizure group (1.71 ± 0.11 ms) compared to sham group (1.91 ± 0.08 ms). In contrast, an increase in the amplitude of fast afterhyperpolarization was observed in GCs of seizure group (-11.68 ± 0.72 mV) compared to sham group (-8.28 ± 0.59 mV). Also, GCs of seizure group showed a significant increase in both firing rate and instantaneous firing frequency at depolarizing currents of 200 pA (P < 0.01) and 250 pA (P < 0.05) compared to sham group. The changes in electrophysiological properties of GCs were attenuated after bath application of paxilline suggesting possible involvement of large conductance Ca^2+^- activated K^+^ channel (BK channel). Our results suggested the possible involvement of certain potassium channels in early changes of intrinsic properties of GCs which eventually facilitate TLE development.

## Introduction

Temporal lobe epilepsy (TLE) is the most common form of acquired epilepsy in adult and is often resistant to antiepileptic drug-therapy (1). In animal models, TLE is a situation that is induced after injection of pilocarpine (2), kainic acid (3), or electrical stimulation of a specific site of brain (4,5) and is associated with recurrent spontaneous seizures. Pilocarpine is a potent M1 muscarinic agonist and its systemic injection to rodents induces behavioral and electrophysiological changes in three distinct phases including acute phase which is caused by an initial brain insult and lasts 24 h, a latent phase that is a relatively seizure-free period and lasts between 4-44 days and a chronic phase in which spontaneous recurrent seizure occurs (6). After a brain insult, a cascade of brain reorganization events termed epileptogenesis is induced which lead to changes in brain excitability and occurrence of spontaneous recurrent seizures (7). Dentate gyrus granule cells (GCs) has long been recognized as a focal point for studies on mechanisms responsible for epileptogenesis. In recent years, much attention has been focused on changes in the intrinsic properties of neurons especially the role of K^+^ channels as a possible mechanism in epileptogenesis and generation of hippocampal epilepsy. For instance, Brenner and coworkers in 2005 reported that β4 subunit of large conductance Ca^2+^ activated K^+ ^channels (BK channels) reduces excitability of dentate gyrus GCs preventing temporal lobe seizures (8). 

Despite many studies on hippocampal seizure, there is little information regarding early events that initiate epileptogenesis in dentate gyrus. Identification of early alterations in electrophysiological properties of GCs would enable us to know how these changes might contribute to the epilepsy. In this study, we evaluated changes in the intrinsic properties of GCs during acute phase of pilocarpine-induced seizure using whole cell patch clamp recordings. 

## Experimental

*Animals*

Male wistar rats weighting 150-200 g (Pasteur Institute, Tehran, Iran) were used in this study. The animals were housed in colony cages (5 rats per cage) with free access to food and tap water under standardized housing conditions with a 12 h light–dark cycle (lights on at 7:00 a.m.) and temperature-controlled (22 ± 1 °C) environment. All procedures were in accordance with the National Institute of Health Guide for the Care and Use of Laboratory Animals (NIH Publications No. 80-23, revised 1996) and were approved by the local Research and Medical Ethics Committee.

*Drugs*

Rats were treated with pilocarpine (350 mg/Kg, *ip*; Sigma-Aldrich Co. St Louis, USA) 20 min after methyl scopolamine (5 mg/Kg, S.C; Sigma-Aldrich Co. St Louis, USA) administration in order to reduce the peripheral effects of pilocarpine. Diazepam (4 mg/kg, *ip*; Sigma-Aldrich Co. St Louis, USA) was administered after 3 h to stop status epilepticus (SE). Only motor seizures of grade 3 or greater on the Racine scale (9) were scored.

*Patch-clamp recordings in hippocampal slices*

*Slice preparation*

Twenty-four hours after seizure induction, rats were anaesthetized with ether and then decapitated. The brains were quickly removed and chilled in ice-cold slicing solution containing (in mM): 125 NaCl, 2.8 KCl, 1 CaCl2, 1 MgCl2, 2 MgSO4, 1.25 NaH2PO4, 26 NaHCO3, 10 D-glucose and set to pH of 7.4 (with 95% oxygen and 5% carbon dioxide); the osmolarity was adjusted to 305 mOsm by addition of sucrose to the solution. The brain transverse slices containing hippocampal area were cut into 350-400 μm using a vibroslicer (752 M, Campden Instruments Ltd, UK). The slices were then incubated in ACSF containing (in mM): 124 NaCl, 2.8 KCl, 2 CaCl2, 2 MgSO4 ,1.25 NaH2PO4, 26 NaHCO3, and 10 D-glucose at pH 7.4, the osmolarity of 295 mOsm and temperature of 32-35 °C for 1h and stored at 22–24 °C (room temperature) before being transferred to the recording chamber.

*Electrophysiology*

The slices were transferred to a submerged recording chamber and were continuously perfused with ACSF (1–2 mL/min) at room temperature. Dentate gyrus GCs were visualized by infrared videoimaging (Hmamatsu, ORSA, Japan) with a 40x water immersion objective. Recordings were made using glass electrodes pulled with a two-stage vertical puller (PC10, Narishige, Japan) from borosilicate glass capillary (1.2 mm O.D., 0.95 mm I.D.). The pipettes had a resistance of 3-6 MΩ and filled with intracellular solution containing (in mM) 135 potassium methylsulfate (KMeSO4), 10 KCl, 10 HEPES, 1 MgCl2, 2 Na2ATP, and 0.4 Na2GTP. The pH of internal solution was adjusted to 7.3 by KOH, and the osmolarity was set to 295 mOsm. Whole-cell patch-clamp recordings were made from dentate gyrus GCs using Multiclamp 700B amplifier (Axon Instruments, Foster City, CA) equipped with Digidata 1320 A/D converter (Axon Instruments, Foster City, CA). Recordings were only obtained when seals of more than 1GΩ resistance were established. The function of test seal was frequently checked during the experiment to ensure that the seal is stable. Access resistance was less than 20 MΩ and less than 20% change during recordings was acceptable in order to include the recording for further analysis. In addition, only cells with resting membrane potential (RMP) more hyperpolarized than -70 mV, input resistance (Rin) >200 MΩ, and obvious overshoot of action potential (AP) were included in analysis. In certain recordings, paxilline (1 μM; Sigma-Aldrich Co. St Louis, USA) was included in bath solution (10), and 1,2-Bis (2-amino-5-bromophenoxy) ethane-*N*,*N*,*N*′,*N*′-tetraacetic acid (BAPTA; 10 mM) was applied in the pipette solution . Electrophysiological recordings were sampled at 10 kHz, filtered at 5 kHz and stored for offline analysis. To investigate the electrophysiological properties of GCs in current clamp mode, trains of APs were elicited by applying depolarizing currents (50-250pA; 1000 ms) while the cell was hold at -75 mV. AP characteristics were measured based on the first AP elicited by the depolarizing current of 200pA (11). Passive and active electrophysiological parameters including RMP, Rin, the number of APs, fast after-hyperpolarization (fAHP) amplitude, AP duration at half-width, decay time, instantaneous firing frequency (IFF) were measured. Rin was defined by the steepest slope of the I-V curve based on steady-state responses to hyperpolarizing current pulses (50–200 pA, 300 ms). The AHP amplitude was measured from the level of RMP to the peak of the hyperpolarization. AP half-width was measured before and 15 min after bath application of paxilline to ensure that maximum effects were obtained. IFF was measured as 1/interval between the first and the second AP. 

*Statistical analysis*

Data were shown as mean ± S.E.M. The Student›s unpaired t-test, one-way ANOVA and two-way ANOVA followed by Bonferroni’s post-test were used as appropriated. A p-value less than 0.05 were considered statistically significant.

## Results

The passive membrane properties of GCs, such as RMP and Rin did not affect by pilocarpine-induced seizure nor by Paxilline application (data not shown). 

*Paxilline reversed hyperexcitability of dentate gyrus GCs in acute phase of pilocarpine-induced seizure*

**Figure 1:**
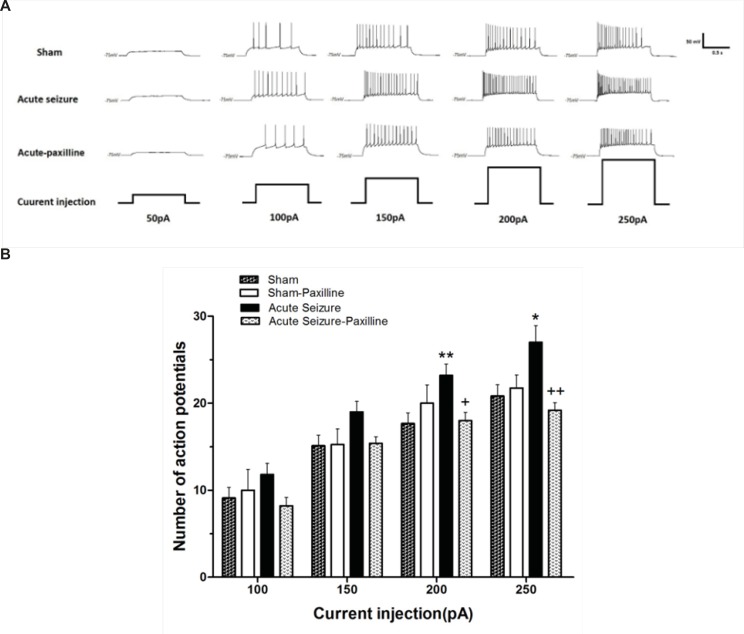
Alteration in the firing rate of dentate gyrus GCs recorded 24 h after pilocarpine-induced seizure. (A) Representative traces show the differences in firing rate of the GCs in response to 1000 ms depolarizing pulses from 50 pA to 250 pA in sham (above), acute seizure (middle) and acute seizure + paxilline (down) groups. (B) Depolarizing current injection ranging from 50 pA to 250 pA increased the number of APs in seizure group compared to sham group. The application of paxilline (1 μM) reversed pilocarpine-induced increase in APs. Data were shown as mean+SEM (N = 10).

The excitability of GCs during acute phase of seizure was evaluated by measuring the number of APs elicited by depolarizing current pulses ranging from 50 to 250 pA. Figure 1A showed traces of APs. Injection of depolarizing currents into granule cells significantly increased the number of action potentials in acute seizure group compared to sham group [F (1, 72) = 23.06, p < 0.0001; Figure 1B]. Further analysis using Bonferroni’s post-test revealed a significant increase in number of APs at 200 (p < 0.01) and 250 pA (p < 0.05) compared to sham group. Bath application of paxilline significantly attenuated seizure-induced increase in APs at 200pA (p < 0.05) and 250 pA (p < 0.01) compared to seizure group. 

*p<0.05, **p<0.01 significant difference compared to sham group.

+p < 0.05, ++p<0.01significant difference compared to acute seizure group.

*Changes in fAHP and AP half-width during acute phase of pilocarpine-induced seizure were reversed by paxilline*

**Figure 2:**
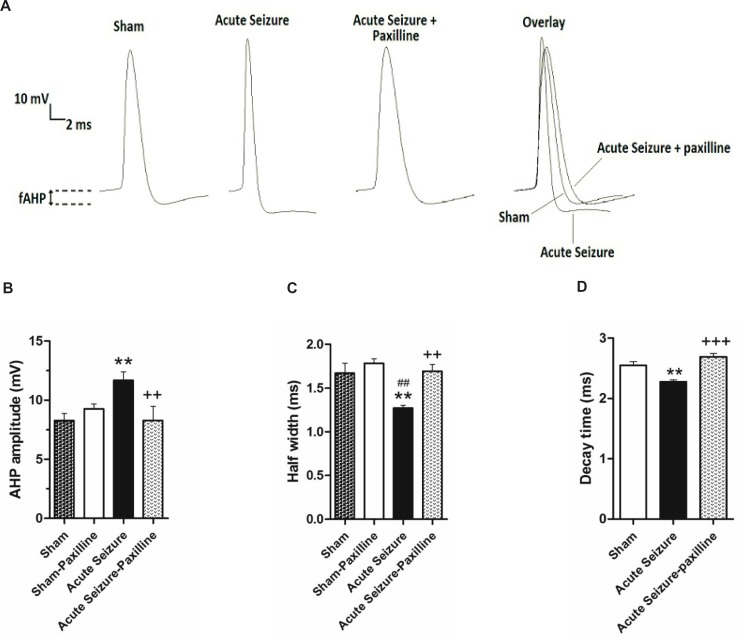
Pilocarpine-induced changes in AHP amplitude and AP half-width were reversed by bath application of paxilline. (A) Representative trace of 1st AP of sham, acute seizure and acute seizure + paxilline groups during a train of AP evoked by 200 pA current injection for 1000 ms. (B) fAHP amplitude was measured from the level of RMP to the peak of the hyperpolarization. fAHP amplitude significantly increased during acute phase of pilocarpine-induced seizure compared to sham group. Paxilline decreased fAHP amplitude to the sham values. (C) AP half- width significantly decreased during acute phase of seizure. Paxilline increased AP half-width to normal values and reversed the effect of seizure on AP half-width. (D) The decay time of AP was increased in pilocarpine-induced seizure group and was reversed after bath application of paxilline.

In order to further illustrate the mechanisms involved in regulation of GCs firing rate, fAHP and half-width of the first AP were measured after injection of the depolarizing current pulse of 200pA. Figure 2A shows a representative trace of the firs AP in sham, acute seizure, and seizure-paxilline group. As shown in Figure 2B, the fAHP of the first AP was significantly changed in different groups [F (3, 25) = 4.48; p = 0.012]. Further analysis revealed a significant increase (p < 0.01) in fAHP of cells in seizure group (-11.67 ± 0.72 mV; N = 8) compared to sham group (-8.28 ± 0.59 mV; N = 8). Bath application of paxilline, significantly reversed seizure-induced increase in fAHP amplitude (p < 0.01) toward sham group (-9.26 ± 0.42 mV, N = 8). Moreover, as shown in Figure 2C, a significant change was observed in the half-width of AP in different groups [F (3, 23) = 6.605; p = 0.002]. Further analysis revealed a significant decrease (p < 0.01) in the half-width of the first AP in seizure group (1.27 ± 0.03 ms; N = 7) compared to sham group (1.60 ± 0.11 ms; N = 8). Decrease in AP half-width of seizure group could be due to decrease in decay time of AP (Figure 2D), suggesting the role of K^+^ channels in reduction of half-width. Bath application of paxilline, significantly reversed (p < 0.01) seizure-induced changes in the half-width of AP toward sham group (1.69 ± 0.08 ms; N = 8).

*p<0.05, **p<0.01 significant difference compared to sham group.

++p<0.01, +++p<0.001 significant difference compared to acute seizure group.

##p<0.01 significant difference compared to sham + paxilline group.

*Application of BAPTA increased half width to normal values*

**Figure 3:**
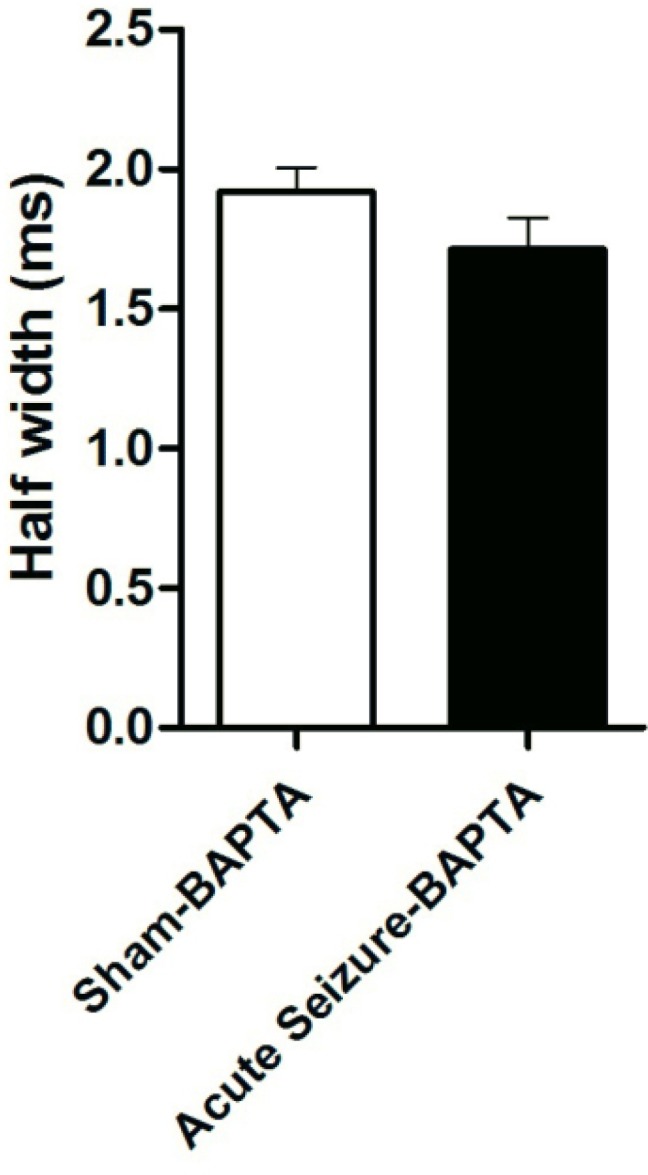
Effect of BAPTA on AP half-width. Application of BAPTA prevented change in AP half-width in seizure group compared to that of BAPTA-sham cells. Data were shown as mean + SEM (N = 5).

The Ca^2+^-activated K^+ ^channels are activated by both membrane depolarization and increase in intracellular Ca^2+^ concentration [Ca^2+^]_i_ (12,13). In order to examine the role of [Ca^2+^] in firing rate of GCs, the Ca^2+^ chelator BAPTA (10 mM) was added to the internal solution from both sham and acute seizure group. Application of BAPTA prevented change in AP half-width in seizure group (1.71 ± 0.11 ms; N = 5) compared to that of BAPTA-sham cells (1.91 ± 0.08 ms; N = 5), suggesting a role of intracellular Ca^2+^ in firing frequency (Figure 3).

*Increase in IFF after seizure is reversed by paxilline application*

**Figure 4:**
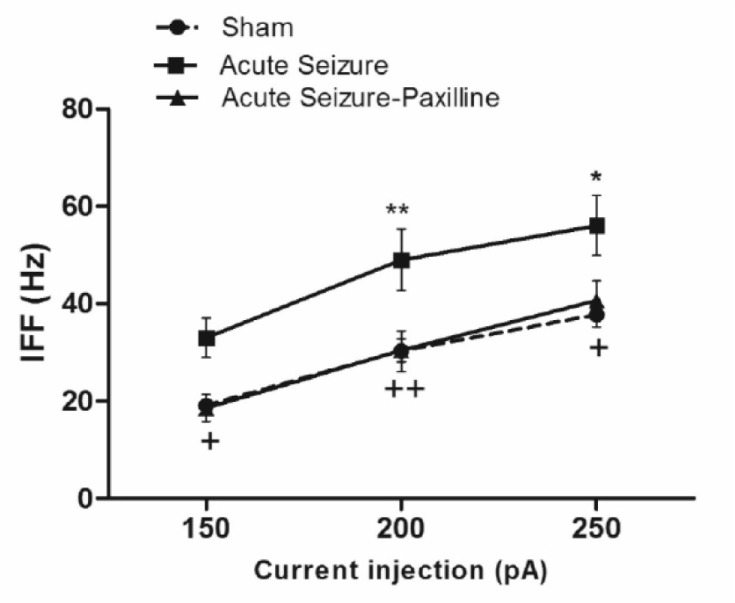
Seizure-induced increase in instantaneous firing frequency (IFF) was reversed by paxilline application. IFF was measured as the reciprocal of interval between the first and the second AP during 200 pA current injection. IFF significantly increased during acute phase of pilocarpine-induced seizure. After bath application of paxilline, IFF returned to sham levels. Data were shown as mean ± SEM (N = 6 in each group).

As shown in Figure 4, IFF was significantly enhanced after seizure [F (2, 39) = 15.94, P < 0.0001]. Further analysis using Bonferroni’s post-test revealed a significant increase in IFF at 200 pA (P < 0.01) and 250 pA (P < 0.05) compared to sham group. Bath application of paxilline significantly reduced IFF to sham values at 150 pA (P < 0.05), at 200 pA (P < 0.01), and 250 pA (P < 0.05), compared to those of sham group, suggesting the possible role of BK channel in enhancement of the firing rate.

*p<0.05, **p<0.01 significant difference compared to sham group.

+p<0.05, ++p<0.01 significant difference compared to acute seizure group.

## Discussion

There are several documents that show the role of dentate gyrus in epileptogenesis (14). Although seizure-induced changes in hippocampal neurons have been studied extensively, the mechanisms that initiate epileptogenesis have not been fully established. In this study, we focused on intrinsic membrane properties of granule cells 24 h after seizure induction and demonstrated that pilocarpine-induced seizures altered intrinsic membrane properties of the dentate gyrus GCs. Pilocarpine caused a significant increase in the firing frequency, the fAHP amplitude, and the IFF while caused a significant reduction in AP half-width and decay time in GCs during acute phase of seizure. Previous studies reported that pilocarpine-induced seizure induces hyperexcitability of hippocampal cells during early stages of epileptogenesis. Using field potential recording, a hyperexcitability, as a transient increase of the input and output field responses, has been shown during the latent period of epileptic animals which may participate in development of epilepsy (15). In other study, patch-clamp recording from GCs in dentate gyrus revealed that the stimulation of perforant pathway produce hyperexcitability of GCs as an increase in the number of action potentials (16). In our study, for the first time, a significant change in certain intrinsic properties of GCs was reported which could result in hyperexcitability of these cells. Also, our results showed that bath application of paxilline attenuated the increase in firing rate of GCs to normal values and reversed the effects of pilocarpine on fAHP amplitude, AP half-width, decay time and IFF, suggesting the role of K^+^ channels, including BK channels, in hyperexcitability of GCs during acute phase of TLE. The BK channels are widely expressed in CNS and are gated both by voltage and by intracellular Ca2+ ions. These channels not only contribute to action potential repolarization and shape the fAHP (17,18), but can also affect neuronal firing patterns (19,20). A relation between seizure and gain-of-function of BK channel has been associated with high firing rate of neocortical neurons with an increase in the AHP amplitude and a decrease in AP half-width (21). Moreover, a gain- of-function of BK channel activity in genetic epilepsy both in human and mice have been associated with recurrent seizures (22,8). The changes in neuronal excitability has been shown in other conditions related to synaptic plasticity, such as learning, where it could be modulated by changing the amplitude of AHP (23). BK channels are one of the most prominent ion channels which have been shown to be involved in the generation of the fAHP (24). The mechanism by which BK channel activity increases the firing rate might contribute to a faster repolarization and a more deinactivation of Na^+^ channels that occurs during the fAHP, increasing Na^+^ channel availability and this resulted in firing with short latencies (19). The observed changes in the intrinsic properties of GCs is likely attributed to [Ca^2+^]_i_, as the elimination of intracellular Ca^2+^ using BAPTA reversed the decreased half-width of APs during acute phase of TLE. Consistent with our results, recent studies have shown an increase of [Ca^2+^]_i_ in rat hippocampal CA1 neurons during the acute phase of pilocarpine model of seizure (25,26) which could induce gain-of-function of BK channels. 

In conclusion, these results suggest that pilocarpine-induced hyperexcitability in dentate gyrus GCs during acute phase could result from alterations in the intrinsic properties of the cells, particularly those related to potassium channels activation which could give rise to an increase in the firing rate of GCs. Considering the possible role of BK channel activity in early stages of epileptogenesis, the blockade of these channels might have a potential therapeutic effect in prevention of synaptic plasticity required for recurrent seizure occurrence.
